# Reply to Kornfeld and Titus: No distraction from misconduct

**DOI:** 10.1073/pnas.1918001116

**Published:** 2019-12-10

**Authors:** Kathleen Hall Jamieson, Marcia McNutt, Veronique Kiermer, Richard Sever

**Affiliations:** ^a^Annenberg School for Communication, University of Pennsylvania, Philadelphia, PA 19104;; ^b^National Academy of Sciences, Washington, DC 20001;; ^c^Public Library of Science, San Francisco, CA 94111;; ^d^Cold Spring Harbor Laboratory, Cold Spring Harbor, NY 11724

Kornfeld and Titus (1) argue that we (2) deceive ourselves by focusing on signaling adherence to scientific norms rather than on perpetrators of scientific misconduct. This is not the case. We explicitly advocate that funders make research ethics a condition of support; that institutions provide education and investigate misconduct fairly, rapidly, and transparently while protecting whistleblowers; that journals act quickly to correct the record; and that spanning organizations such as the National Academies establish norms and arbitration mechanisms.

Our core contention is that scientists and the outlets that publish their work should not only honor science’s integrity-protecting norms but also clearly signal when, and how, they have done so. Many of the interventions that serve those ends (including the use of checklists, badges, statistical checks, plagiarism checks, ORCIDs, forward linking, an improved withdrawal ontology, and more complete declaration of competing interests) help detect and discourage cheating. At the same time, they help uncover and increase awareness of biases that can undermine researchers’ ability to fairly interpret their findings. Significantly, these indicators of trustworthiness clearly signal that the scientific community is safeguarding science’s norms and institutionalizing practices that protect its integrity as a way of knowing.
